# Wheelchair service provision training during armed conflict: preliminary results from a pre-post study in Ukraine

**DOI:** 10.3389/fresc.2025.1723913

**Published:** 2025-12-15

**Authors:** M. Tofani, V. Golyk, K. Dieieva, A. Kamadu, M. E. Quinn, A. E. Tawashy

**Affiliations:** 1 Rehabilitation and Disability Unit, World Health Organization Country Office of Ukraine, Kyiv, Ukraine; 2 Department of Life Sciences, Health and Healthcare Professions, Link Campus University, Rome, Italy; 3 International Society of Wheelchair Professionals, Pittsburgh, PA, United States; 4 School of Occupational Therapy, Dalhousie University, Halifax, NS, Canada

**Keywords:** armed conflict, assistive technology, rehabilitation capacity-building, rehabilitation professionals, wheelchair service training

## Abstract

**Background:**

Universal Health Coverage (UHC) cannot be achieved without equitable access to assistive technology (AT). Wheelchairs are among the most needed AT products worldwide, yet service provision is hindered by limited workforce capacity, inadequate training, and fragile supply systems, challenges that become critical in conflict and emergency settings.

**Objective:**

This study aimed to evaluate the effectiveness of a World Health Organization (WHO)- supported wheelchair service training program in Ukraine, developed in partnership with the International Society of Wheelchair Professionals (ISWP), in improving theoretical knowledge, wheelchair skills performance and confidence, among rehabilitation professionals

**Methods:**

A five-day, 40 h training program based on the WHO Wheelchair Service Training Package–Basic Level (WSTPb) was delivered to 39 rehabilitation professionals in Ukraine. Training combined theoretical instruction, hands-on skill practice, and adapted educational strategies, including group-based ISWP testing, to overcome infrastructure constraints. Pre- and post-training assessments were conducted using the Wheelchair Skills Test–Questionnaire (WST-Q).

**Results:**

All participants achieved certification in basic wheelchair service provision. Statistically significant improvements were observed in both performance (from 42.72 ± 21.89 to 68.08 ± 14.22; mean increase 25.36%) and confidence (from 40.72 ± 21.63 to 67.72 ± 12.88; mean increase 27.00%) domains of the WST-Q (*p* < 0.01). Importantly, rehabilitation assistants demonstrated the largest relative improvement, reducing pre-training disparities with occupational and physical therapists.

**Conclusion:**

The findings highlight how targeted educational interventions can expand the AT workforce, promote equitable skill acquisition across professional cadres, and strengthen AT integration into UHC, even in the context of armed conflict. The Ukrainian experience illustrates both the clinical challenges, such as mastering advanced wheelchair skills, and the educational challenges including addressing diverse professional backgrounds and limited infrastructure, that are inherent in AT service provision. This model can inform future workforce capacity-building strategies for AT in both emergencies and routine health system strengthening efforts.

## Introduction

For people with mobility impairments, an appropriate wheelchair is essential for health and wellbeing (1), but also enhances fundamental rights, and guarantees equal opportunity and participation in society. Using an appropriate wheelchair supports the right to personal mobility enshrined in the UN Convention on the Rights of Persons with Disabilities (2).

The World Health Organization (WHO) estimates that 10% of people with disabilities worldwide need a wheelchair, but only 5%–15% of them have access to one (3). This means that about 95 million people do not have a wheelchair at all or have one that is inappropriate for their needs. The main problems due to inadequate service provision include low-quality products, inefficient provision, lack of qualified provision personnel, and inadequate policies (4). In emergency situations, such as war- related conflicts, these issues are particularly highlighted, and multi-stakeholder partnerships are urgently required to deal with them.

Before the invasion by Russia of Ukraine on 24 February 2022, the WHO Regional Office for Europe and the WHO Country Office in Ukraine were supporting the Ministry of Health of Ukraine (MoH), the Ministry of Social Policy of Ukraine (MoSP) and other state agencies in integrating rehabilitation into the national health system (5). The MoH and MoSP recognized rehabilitation and assistive technologies as key components for universal health coverage and defined strategies to implement services across the country. With the subsequent war, the needs for rehabilitation and assistive technologies (including wheelchairs) dramatically increased. One of the main challenges for appropriate wheelchair provision in Ukraine was the lack of qualified healthcare professionals. Consequently, the MoH and MoSP set a strategic priority to develop the primary healthcare workforce through providing educational opportunities (6).

To overcome the gap of the unmet educational needs worldwide, the WHO published the 2008 Guidelines on the Provision of Manual Wheelchairs in Less Resourced Settings (3). This manual addressed the design, production, supply and service provision for manual wheelchairs, targeting several stakeholders, including policy-makers, service providers, designers and manufacturers, and wheelchair users. In 2012 several packages for wheelchair training were developed: the Basic Level, the Intermediate Level, the Package for Managers and Stakeholders and, the Training of Trainer package. In particular, the Wheelchair Service Training Package—Basic level (WSTPb) (7) is the entry level course designed to support the training of all personnel to provide an appropriate manual wheelchair for children and adults who have mobility impairments but who can sit upright without additional postural support. This course, endorsed by the International Society of Wheelchair Professionals (ISWP) has recently been updated to reflect the current best practice in wheelchair provision. In 2023, the WHO published the Wheelchair Provision Guidelines (8) which were co developed with ISWP and build on the 2008 WHO Guidelines on the Provision of manual wheelchairs in less resourced settings and draw on a growing body of evidence.

Despite the availability of standardized resources such as the WSTPb and the growing global emphasis on strengthening AT provision, the evidence supporting wheelchair service and skills training has been generated almost in stable, non-conflict settings (9, 10); to date, no research has examined whether wheelchair service training is feasible or effective when delivered during armed conflict, where disrupted infrastructure, safety constraints, and heterogeneous professional backgrounds pose unique challenges. This represents a critical gap, particularly as conflict settings dramatically heighten the need for context-adapted, rapidly deployable training strategies to strengthen local workforce capacity. To address this gap, the present study evaluated the effectiveness of a WHO-supported wheelchair service training program in Ukraine—implemented during an ongoing armed conflict—in improving theoretical knowledge, wheelchair skill performance, and confidence among rehabilitation professionals.

## Material and methods

The present investigation was part of a comprehensive WHO project that supports Ukraine to strengthen rehabilitation and assistive technology. This study was approved by Institutional Review Board of Public Health Center Ministry of Health of Ukraine (Protocol number: RB2024-145) and by the World Health Organization Ethics Committee (Protocol number: ERC.0004237).Our working group consisted of three subgroups: (1) two WHO international consultants with an Occupational Therapy background, with more than 10 years of experience in wheelchair service provision and active membership in ISWP; (2) national rehabilitation professionals with knowledge of the Ukrainian context; and (3) interpreters for synchronous English- Ukrainian translation throughout the process. To ensure the accuracy and consistency of all content, the interpreting team consisted of Ukrainian professionals with established experience in medical and rehabilitation terminology, who had previously collaborated with the WHO on rehabilitation-related projects. Their direct involvement in the recent revision of the Ukrainian version of the WSTPb manual meant that they were already well acquainted with the technical language, conceptual framework, and methodological structure of the training. This combination of prior expertise and familiarity with the materials helped minimize translation bias and ensured consistent and faithful translation throughout the training.

### Training course

Although new WHO wheelchair provision guidelines have recently been developed (8), they do not include the detail needed for step-by-step provider training. Therefore, this training course was based on the 8-steps of wheelchair service provision described and updated by the WSTPb. These steps are as follows: Step 1: Referral and appointment; Step 2: Assessment; Step 3: Prescription (selection); Step 4: Funding and ordering; Step 5: Product (wheelchair) preparation; Step 6: Fitting; Step 7: User training; Step 8: Maintenance, repairs and follow up. Because the WSTPb is one of the most widely used training manuals for wheelchair service provision (11), the WHO opted to use the Ukrainian translation and printing of its reference manual and training workbooks.

According to WSTPb recommendations, the training course was organized to take place in 40 h over five consecutive days. The program was adapted to the specific Ukrainian context by considering the target population and the locally available products, and was set-up using the Wheelchair Educators' Package (WEP) methodology (12), promoted by the ISWP. We used the translated WSTPb Manual, adapting the content to the updates from the ISWP review and the 2023 WHO Guidelines on Wheelchair Service Provision (8). Of note, we had a particular focus on Fit (Step 2) and Training (Step 3), using the local vendors to provide specific training on modifying the locally available products, and the Wheelchair Skill Program methodology and resources (13), respectively.

The training was delivered in the Rivne region, specifically at the National Spinal Cord Injury Center in Klevan. This location was selected in coordination with WHO and local authorities, as western Ukraine has remained comparatively safer during the ongoing armed conflict, with active hostilities concentrated in the eastern and southern regions. This ensured participant and trainer safety and allowed uninterrupted course implementation.

The training followed a structured progression that combined theoretical lectures, group-based work, and supervised practical sessions. Participants rotated across small-group stations to practice with locally available wheelchair models, alternating roles as providers and user to consolidate step-specific competencies. In addition, participants worked in small groups (4–5 persons) with inpatients of the rehabilitation hospital, applying the full 8-step process under supervision; at the end of this activity, each wheelchair user received a newly fitted wheelchair in accordance with ISWP-WHO principles.

For wheelchair skill training, we dedicate a mean of 12.5 h out of 40. This allocation was intentional and based on two considerations. First, an extended practical component was necessary to ensure that participants could acquire the ability to perform wheelchair skills with sufficient consistency to support user training. This competency requires supervised practice and cannot be achieved through theoretical instruction alone. Second, dedicating one-third of the program to practical skill acquisition allowed us to balance theoretical content with practical activities, which is consistent with educational principles recommended in the Wheelchair Skills Program and with best practices for adult learning.

### Participants

Training participants were rehabilitation professionals—employees of rehabilitation departments at municipal hospitals from different oblasts of Ukraine, nominated by leadership of departments/hospitals. Chosen participants worked as either a Physical therapist (PT), Ergotherapist (Ukrainian name of Occupational therapist, OT) or Assistant of Physical therapist (APT) or Assistant of Ergotherapist (AOT). At the time of enrollment, participants were informed of the methods and objectives of the project.

We opted to divide participants into two groups and organize two twin courses in two consecutive weeks. This allowed us to guarantee an appropriate ratio between trainers and trainees (1:10) and to organize adequate practical activity. For the assessment of knowledge, the ISWP Basic Test was administered post-training, while for wheelchair skill examination specific assessment tools were administered pre- and post-training.

### Assessment tools and analysis

#### ISWP basic test

The ISWP Basic Test was launched in 2015. Developed as an online multiple-choice test, it included 75 items taken from a total pool of 145 items (14). Items were aligned with the content of the WHO 8-steps of wheelchair service delivery (7), but they were grouped differently. A total percentage of test scores of at least 70% is considered a passing grade. However, considering the specific Ukrainian context, the heterogeneity of participants and lack of personal computer for each participant, as well as poor internet connection, the research group—with ISWP permission—opted to print-out the full set of 145 questions, to complete in learning groups of 4 people and then evaluate and debrief in larger group of 20 persons. Therefore, we used ISWP Basic Test as a training technique and approach to clarify doubts and guarantee proper learning. Considering this new approach, the ISWP Basic test was registered as a dichotomic variable, categorized as passed/failed according to the percentage of correct answers (≥70%).

#### Wheelchair skill test—questionnaire (WST-Q)

The 5.4 version of the WST-Q (15) is an outcome measure originally developed for wheelchair users and their caregivers to evaluate their self-perceived ability to perform specific wheelchair skills. It includes 30 items, assessing indoor (item 1–10 and 13) community (item 11–12 and 14–21) and advanced (item 22–30) wheelchair skills. The WST-Q allows to rate how well wheelchair user can perform each skill, how confidently and how frequently. Scoring is from 0 to 3 and higher scores mean best performance, confidence and higher frequency. For *Performance* questions participants are asked to rate how well and safely they perform a skill, while for *Confidence* questions they are asked to score how they are feeling confident in performing a skill safely and consistently. Total percentage scores were calculated. Although originally developed for wheelchair users, the WST-Q has been used in several educational studies involving rehabilitation professionals or students (16, 17), where it functions as a proxy indicator of their perceived competence and confidence in executing wheelchair skills, an essential prerequisite for safely teaching, demonstrating, and spotting these skills during clinical practice. For this reason, in the present study we used the WST-Q to assess rehabilitation professionals' perceived performance and confidence, and only these two sections were administered. The WST-Q was administered before starting (T0) and after training (T1); to calculate differences in pre-post training, first we calculate test for normality distribution using Shapiro–Wilk test, and then we applied a parametric or non-parametric test, accordingly, analyzing mean (SD) or median (IQR) scores in both performance and confidence questions. Two primary outcomes were specified *a priori* (performance and confidence). To account for the two comparisons, a Bonferroni-adjusted alpha level of 0.025 (0.05/2) was applied, and statistical significance was set for a *p* < 0.025 (95% CI). Effect sizes were calculated using Cohen's *d* and Hedges' *g*. Cohen's *d* quantifies the magnitude of change between pre- and post-training scores by expressing the mean difference in units of pooled standard deviation. Hedges' *g* provides an adjusted estimate of effect size that corrects for small sample bias and is therefore more appropriate for studies with fewer than 50 participants, such as the present one. For both indices, values of 0.2, 0.5, and 0.8 or grated are conventionally interpreted as small, moderate, and large effects, respectively.

## Results

**Figure 1:**
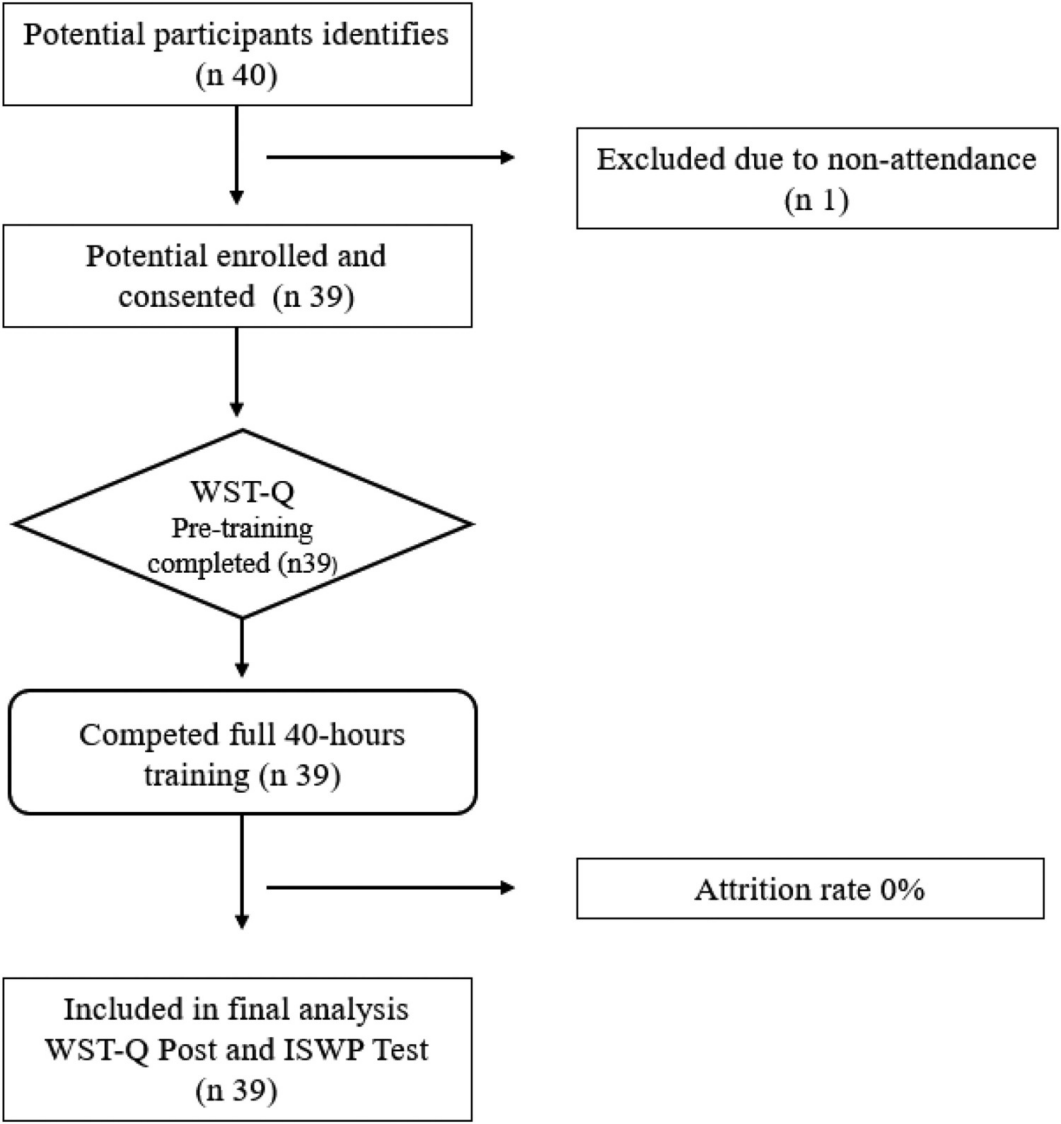
Flow-diagram of selection, attrition and completion of the training.

A total of 40 participants were selected. From the original 40 selected participants, one person could not attend the training, so a total of 39 people participated in the course. Table 1 reports sociodemographic characteristics of participants, while Figure 1 illustrates recruitment, attrition and completion of the training.

**Table 1:** Sociodemographic characteristics of participants.

Variable	Value
Age	Mean (SD)
	29.01 (9.68)
Age range	18–57
Gender	*N* (%)
Male	19
Female	20
Years of experience	*N* (%)
<1 year	22 (58%)
>3 years	8 (21%)
Profession	*N* (%)
PT-OT	24 (61.54)
APT-AOT	15 (38.46)

After the training, all participants reached the 70% of correct answers and therefore passed the ISWP Basic Test for knowledge on wheelchair service provision. For wheelchair skills, we reported data for both pre- and post-training of the WST-Q items for Performance and Confidence domains. From a qualitative point of view, data highlighted that item 24–30 showed lower scores for both confidence and performance questions. Descriptive analysis is synthetized in Table 2.

**Table 2:** Pre- post-training scores of WST-Q performance and confidence domains.

Item	Performance								Confidence							
	Pre				Post				Pre				Post			
	Min	Max	Mean	SD	Min	Max	Mean	SD	Min	Max	Mean	SD	Min	Max	Mean	SD
1	1	3	2.62	.59	2	3	2.97	.16	1	3	2.59	.67	2	3	2.97	.16
2	1	3	2.51	.60	2	3	2.92	.27	1	3	2.44	.71	2	3	2.92	.27
3	0	3	2.15	1.09	2	3	2.85	.36	0	3	2.03	1.08	2	3	2.87	.33
4	0	3	1.92	1.03	2	3	2.79	.40	0	3	1.92	1.03	2	3	2.82	.38
5	0	3	1.59	1.06	1	3	2.67	.57	0	3	1.51	.99	1	3	2.67	.57
6	0	3	1.64	1.11	1	3	2.59	.63	0	3	1.62	1.09	1	3	2.77	.48
7	0	3	2.13	.92	2	3	2.90	.30	0	3	2.05	.88	1	3	2.85	.43
8	0	3	2.05	1.11	2	3	2.92	.27	0	3	1.97	1.07	2	3	2.90	.30
9	0	3	1.97	1.11	1	3	2.64	.53	0	3	1.67	1.08	1	3	2.69	.52
10	0	3	1.76	1.05	0	3	2.66	.66	0	3	1.64	1.08	1	3	2.76	.49
11	0	3	1.59	1.14	1	3	2.64	.53	0	3	1.59	1.09	1	3	2.69	.52
12	0	3	1.82	.99	1	3	2.72	.51	0	3	1.72	.99	1	3	2.62	.59
13	0	3	1.87	.97	2	3	2.79	.40	0	3	1.77	1.01	1	3	2.72	.51
14	0	3	1.44	1.04	1	3	2.51	.55	0	3	1.36	1.11	1	3	2.46	.60
15	0	3	1.46	1.04	1	3	2.56	.55	0	3	1.38	1.04	1	3	2.51	.60
16	0	3	1.33	1.24	0	3	2.38	.74	0	3	1.08	1.13	1	3	2.41	.71
17	0	3	1.18	1.14	1	3	2.46	.64	0	3	1.05	.99	1	3	2.49	.60
18	0	3	1.26	1.01	1	3	2.38	.63	0	3	1.23	1.03	1	3	2.38	.63
19	0	3	1.28	1.07	1	3	2.31	.73	0	3	1.18	.97	1	3	2.33	.62
20	0	3	1.41	1.01	0	3	2.31	.76	0	3	1.36	1.06	1	3	2.41	.67
21	0	3	1.44	1.07	0	3	2.31	.76	0	3	1.33	.98	0	3	2.26	.78
22	0	3	.90	.94	0	3	1.64	1.06	0	3	.97	.95	0	3	1.64	.93
23	0	3	.90	.94	0	3	1.67	1.01	0	3	.81	.93	0	3	1.62	.99
24	0	3	1.18	1.18	0	3	2.05	.94	0	3	1.08	1.10	0	3	1.92	.95
25	0	3	1.05	1.07	1	3	2.13	.83	0	3	1.05	1.09	1	3	2.03	.81
26	0	3	.77	.98	0	3	1.64	1.08	0	3	.74	.96	0	3	1.64	1.08
27	0	3	.44	.85	0	3	1.05	1.07	0	3	.51	.88	0	3	1.05	1.05
28	0	3	.38	.74	0	3	1.05	1.05	0	3	.38	.71	0	3	.95	.97
29	0	2	.36	.58	0	3	.77	.81	0	2	.36	.53	0	2	.69	.73
30	0	2	.41	.59	0	3	.85	.90	0	2	.41	.54	0	3	.74	.85

The Shapiro–Wilk test revealed normal distribution for both Performance (*W* = 0.965, *p* = 0.268) and Confidence (*W* = 0.966, *p* = 0.285) domains. Therefore, we analyzed differences in mean (SD) scores using a paired sample *t*-test. The total percentage Performance and Confidence scores of the WST-Q from pre- to post- training, showed statistically significant improvements for the whole sample in both Performance and Confidence domains (*p* < 0.01), with a great relative improvement for confidence (27%). Results are synthetized in Table 3.

**Table 3:** Mean (SD) differences in WST-Q pre-post training with effect size.

WST-Q scores	Mean (SD) pre	Mean (SD) post	Mean diff (95%CI)	Sig. (2 tailed)	Cohen's d (paired)	Hedges' g (95%CI)
Performance	42.72 (21.89)	68.08 (14.22)	25.36 (18.14–32.58)	<0.01*	1.10	1.08 (0.70–1.46)
Confidence	40.72 (21.63)	67.72 (12.28)	27.00 (20.03–33.97)	<0.01*	1.22	1.19 (0.82–1.57)

*p < 0.01.

We analyzed differences in scoring in pre- and post-training according to professional background. From a qualitative point of view, it is possible to state that OTs and PTs obtained higher score than APTs and AOTs. The mean (SD) WST-Q confidence scores rose from 49% (21.30) to 69.04 (14.04), a 20.04% relative improvement for PTs and OTs, while the confidence scores rose from 29.69% (17.45) to 65.08% (10.13), a 35.39% relative improvement for APTs and AOTs. The mean (SD) WST-Q performance scores rose from 49% (21.30) to 69.6 (15.27), a 20.69% relative improvement for PTs and OTs, while scores of APTs-AOTs rose from 30.15% (17.75) to 64.85% (15.27), a 34.7% relative improvement. Furthermore, in pre-training a statistically significant difference in both Performance and Confidence were observed (*p* < 0.01), but after training differences were level out for both domains. Data are reported in Table 4.

**Table 4:** Differences in pre- and post-training according to professional background.

WST-Q	Timing	PT/OT	APT AOT	Mean diff (95% CI)	*p*	Cohen's d	Hedges' g (95%CI)
Performance	Pre-	49.00 (21.30)	30.15 (17.75)	18.84 (6.19–31.50)	<0.01*	0.93	0.91 (0.22–1.61)
	Post-	69.69 (15.27)	64.85 (15.27)	4.84 (–3.83–13.52)	0.32	0.34	0.33 (–0.34 to 1.00)
Confidence	Pre-	46.23 (21.69)	29.69 (17.45)	16.54 (3.91–29.17)	<0.01*	0.81	0.79 (0.10–1.48)
	Post	69.04 (14.04)	65.08 (10.13)	3.96 (–3.75–11.68)	0.37	0.31	0.30 (−0.37 to 0.97)

*p < 0.01.

**Figure 2:**
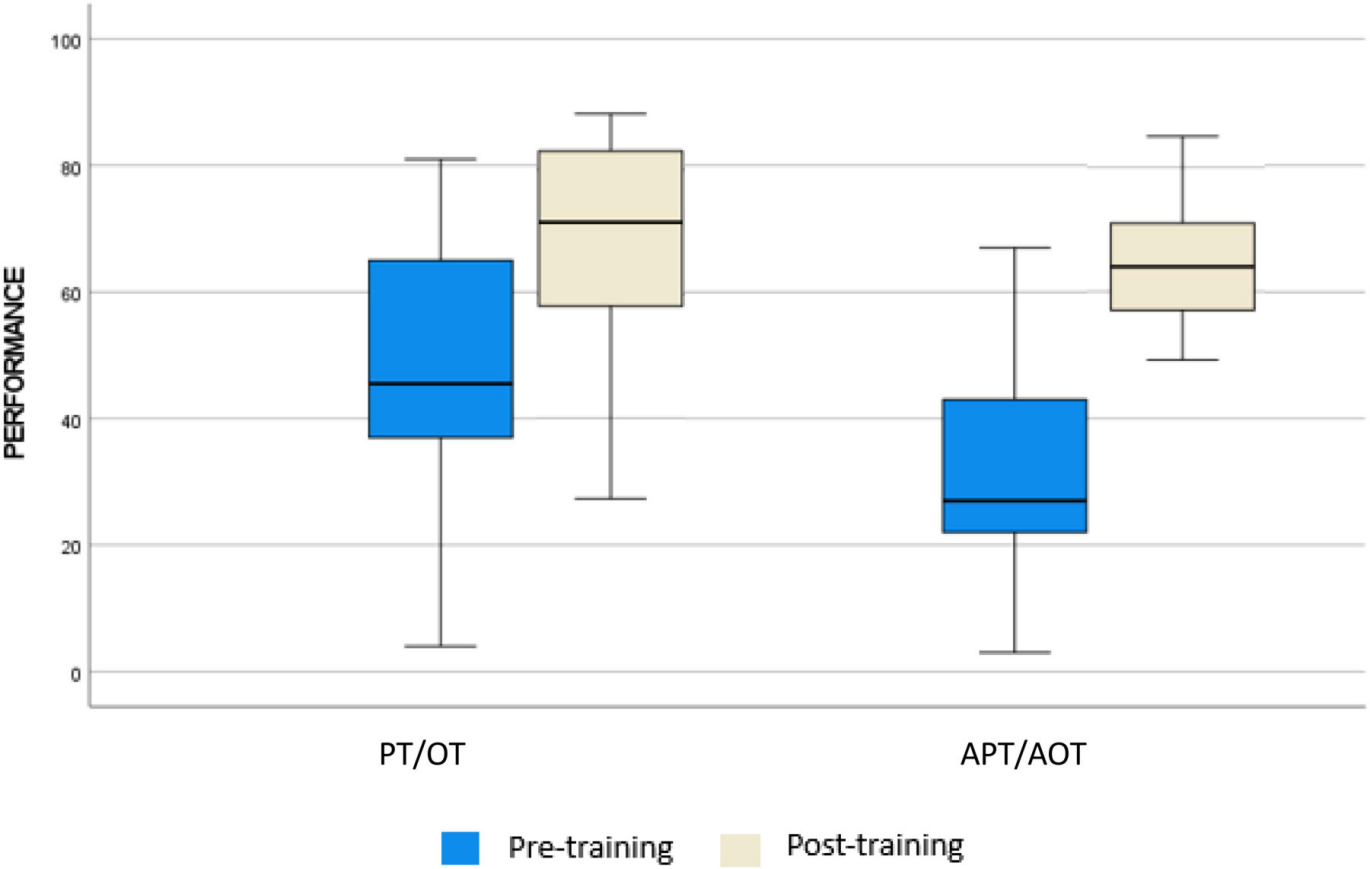
Performance score at WST-Q for physical/occupational therapist and assistants.

**Figure 3:**
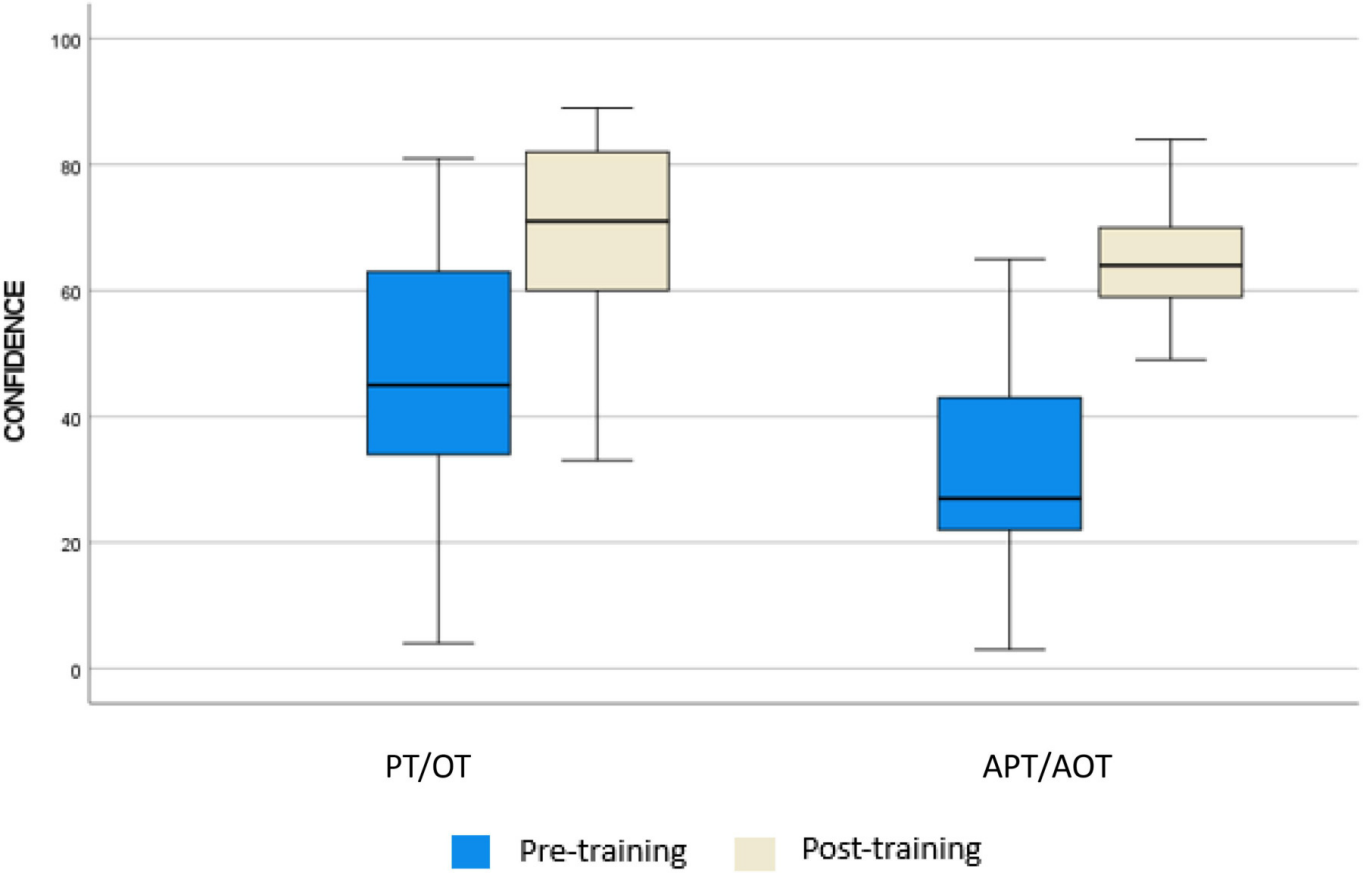
Confidence score at WST-Q for physical/occupational therapist and assistant.

To better understand differences in scoring between PT-OT and APT-AOT across WST-Q domains, results are reported in Figures 2, 3.

## Discussion

The Wheelchair Service Provision training in Ukraine showed positive outcome in terms of both knowledge and practical point of view. All participants passed the ISWP Basic Test and now 39 rehabilitation professionals in Ukraine are certified as wheelchair service providers for the basic level. To the best of our knowledge, and according to ISWP database, to date, they are the first and only rehabilitation professionals to be certified at that level from the ISWP (18).

Our findings also revealed positive and effective outcome in Step 3 (Training) of the new guidelines of wheelchair provision (8); participants showed a significant and statistical improvement (*p* < 0.01) in both total WST-Q performance and confidence domains, with a mean relative improvement 25.36% and 27.00%, respectively, from the baseline to the post-test evaluation. Our results are in line with previous studies in high- and middle-income countries (17, 19, 20) confirming the robustness of wheelchair skills program for developing practical knowledge and confidence in wheelchair skills, regardless of the setting of intervention. Interesting findings were highlighted in differences among education levels of rehabilitation professionals; in particular, despite at the beginning therapists` assistants showed lower score in WST-Q, with statistically significant differences (*p* < 0.01), during the training they obtained a higher relative improvement for both Performance (34.7%) and Confidence (35.39%) that allowed them to reach the approximately the same level of performance and confidence of OTs and PTs (*p* > 0.05). A possible explanation is that therapists began the course with higher baseline scores due to greater prior exposure to wheelchair service delivery, whereas assistants had more room for progression. Their steeper learning curves therefore reflect both baseline differences and the capacity of structured, hands-on training to rapidly elevate the competencies of cadres who traditionally receive less formal preparation in wheelchair provision. This finding is of particular importance, as it suggests that the training was effective regardless of educational background and that assistants may represent a key workforce component for regulated and scalable task-sharing models, especially in health systems facing workforce shortages or instability due to conflict.

As a qualitative point of view, we noticed that performance scores were similar or slightly higher than confidence scores. This is quite similar to findings from several studies conducted in Canada (17, 19, 21). Similar to these authors' reasoning, we thought this to be reasonable as performance it- self is often related to self-confidence in performing a skill. If a higher level of confidence in wheelchair skills among wheelchair users leads to increased mobility and participation (22), similarly one could assume that a higher level of confidence in wheelchair skills among participants may lead to increased attempts to perform wheelchair skills, which would positively affect skill performance. In both groups we found lower scores in performing advanced wheelchair skills (item 22–30 of the WST-Q). This has also been noted in previous studies and aligns with the fact that the construct of WST-Q provides a set of items in a progressive descending order according to difficulty (15, 23, 24). Clinically, this suggests that more time and reinforcement are needed to achieve mastery of complex wheelchair skills, echoing findings from systematic reviews showing that ongoing mentorship and refresher courses are key for consolidating advanced competence (22). Short, intensive training programs, while effective for foundational skills, may therefore be insufficient to develop higher-level competencies. This highlights the need for structured follow-up opportunities, supervised practice sessions, and periodic refresher training to ensure that clinicians can safely perform and teach these advanced skills in their daily practice. It is also important to consider whether the group-based learning format may have contributed disproportionately to the improvement in confidence. Working in small groups, receiving immediate peer feedback, and engaging in shared problem-solving are known to reduce performance anxiety and create a safe learning environment. These elements may have helped participants feel more comfortable when attempting new or challenging skills, thereby enhancing perceived confidence even beyond the level of their actual performance. While this supportive atmosphere is a strength of the training model, it raises the possibility that confidence may have increased more rapidly than demonstrated skill acquisition for some participants.

Despite these encouraging results, there is a need to acknowledge some limitations. First of all, first, the study used a pre–post design without a control group. Although acceptable for preliminary research, this design limits causal inference and may be affected by threats to internal validity. Secondly, the small sample size restricts the statistical power and limits the generalizability of the results. Moreover, no long-term follow-up was conducted, preventing the assessment of knowledge retention over time.

Regarding measurement issues, we measured the multiple-choice ISWP Basic Test as dichotomic variable and as such, we could not analyze data based on domain (wheelchair service steps). Results of this particular analysis may have the potential to better tailor subsequent training courses according to the specific Ukrainian context. We surmised that the multiple choice format of the ISWP Test may not have been an ideal for our course. In our participant sample, we had high variabilities in participant age and participant level of education. While multiple choice tests are common in academia, they may not be encountered frequently in other settings. As such, participants with advancing age (who completed high school >20 years prior) and those who completed basic training programs may not have been as familiar with the specific logic framework that is required for successful completion of this type of test format. As such, we opted to have the participants complete their tests in small groups and we marked them via group discussion. While obtaining individual scores was not possible, we believe that using this approach still ensured a satisfactory level of comprehension among our participants. We acknowledge, however, that the group format may have contributed to higher pass rates compared with individual administration. Furthermore, it is widely noted that for teaching wheelchair skills, there is a need to have self-confidence via good performance in a safe environment. Accordingly, we used the WST-Q to evaluate self-confidence and performance. However, we did not objectively assess the change in participants' confidence or ability in “spotting” wheelchair users (an integral skill for teaching wheelchair skills). Thus, another limit of our study is that this particular construct was not measured. In the future, it would be useful to insert the Self Efficacy on Assessing Training and Spotting (SEATS), a self-report measure of clinicians' self-efficacy to assess, train and spot each of the 33 wheelchair skills on the Wheelchair Skills Test (25). At the end, we used a translated version of the WST-Q, but there is no official version of that outcome measure into Ukrainian with measured psychometric properties; despite we followed international recommendations to guide this process of translation, there are no available data in terms of reliability and validity. Future studies can be directed toward the evaluation of psychometric properties to use for both educational and clinical purposes.

These findings should also be understood in the broader context of Universal Health Coverage. As highlighted in the WHO-UNICEF Global Report on Assistive Technology (4), a trained and adequately distributed AT workforce is essential for achieving UHC, yet remains one of the greatest systemic bottlenecks worldwide. Our results confirm that educational interventions can expand workforce capacity even in resource-limited and crisis contexts, and they align with global evidence on the role of AT training in health system strengthening (26). The fact that rehabilitation assistants, after targeted training, were able to reach competence levels comparable to therapists also demonstrates the potential of structured task-sharing models, which are increasingly promoted within global health policy to address workforce shortages (27). Furthermore, the Ukrainian context, with its dual challenges of war-related disruption and ongoing health reform, exemplifies how flexible and context-sensitive educational strategies—such as group-based testing and adapted curricula—can ensure inclusiveness and effectiveness despite infrastructural limitations.

The present investigation lay the foundation to create a context specific training for wheelchair service provision in war-related conflicts, following international guidance and an evidence-based approach. Considering encouraging results, the working group is planning to organize further training course to train as rehabilitation professional as possible to obtain ISWP basic level certification. As WHO Ukraine strategic vision, leveraging on MoH and MoSP support, several basic training courses are planned, together with a ISWP intermediate level course specific for the Ukrainian context. Furthermore, as highlighted from a recent report from Ukraine (6) there is a need to create a national taskforce of allied healthcare professionals who can strengthen assistive technology sector in the country. In the next future, we are planning a first step of training of trainers to implement in Ukraine for rehabilitation professionals who can serve as local trainer at national level.

In conclusion, this study provides preliminary but robust evidence that a structured and context-sensitive wheelchair service provision training program can substantially improve knowledge, performance, and confidence among rehabilitation professionals, even in the challenging conditions of an ongoing armed conflict. The training effectively reduced baseline disparities between professional profiles, expanded the pool of certified wheelchair service providers, and demonstrated the feasibility of strengthening the assistive technology workforce in crisis-affected settings. Building on these results, future work should include longitudinal evaluations to assess knowledge retention, validation of Ukrainian versions of assessment tools, integration of complementary competency measures and further development of a national training-of-trainers model to ensure sustainability. These steps will help establish a resilient, locally led system for wheelchair service provision in Ukraine and may offer valuable guidance for similar initiatives in other armed conflict-affected contexts.

## Data Availability

The original contributions presented in the study are included in the article/Supplementary Material, further inquiries can be directed to the corresponding author.

## References

[1] Berardi A Galeoto G Lucibello L Panuccio F Valente D Tofani M. Athletes with disability’ satisfaction with sport wheelchairs: an Italian cross sectional study. Disabil Rehabil Assist Technol. (2020) 16:420–4. 10.1080/17483.10720201800114 32730722

[2] United Nations D of E and SAD. Convention on the Rights of Persons with Disabilities (CRPD) | United Nations Enable. (2006). Available online at: https://www.un.org/development/desa/disabilities/convention-on-the-rights-of-persons-with-disabilities.html (Accessed August 27, 2021).

[3] World Health Organization. Guidelines on the Provision of Manual Wheelchairs in Less Resourced Settings. Geneva: WHO (2008). 23785745

[4] WHO-UNICEF. Global Report on Assistive Technology. Geneva: WHO/UNICEF (2022).

[5] Gosling J Golyk V Mishra S Skelton P. We must not neglect rehabilitation in Ukraine. EClinicalMedicine. (2022) 50:101537. 10.1016/j.eclinm.2022.101537 35812991 PMC9257332

[6] WHO. A Situation Assessment of Assistive Technology in Ukraine. Copenaghen: World Health Organization. Regional Office for Europe (2022).

[7] World Health Organization. Wheelchair Service Training Package: Reference Manual for Participans: Basic Level. Geneva: World Health Organization (WHO) (2012).

[8] World Health Organization. Wheelchair Provision Guidelines. Geneva: World Health Organization (2023). 37410877

[9] Sarsak H. Developing wheelchair training program for rehabilitation and occupational therapy students. MOJ Yoga Phys Ther. (2018) 3(4):79–83. 10.15406/mojypt.2018.03.00049

[10] Kirby RL Smith C Rushton P Sandila N. Manual wheelchair-skills courses (“bootcamps”) for wheelchair service providers: an observational study on learners’ satisfaction and perceived effectiveness. Disabil Rehabil Assist Technol. (2025):1–14. 10.1080/17483107.2025.2561928 40990736

[11] Burrola-Mendez Y Kamalakannan S Rushton PW Bouziane S-A Giesbrecht E Kirby RL Wheelchair service provision education for healthcare professional students, healthcare personnel and educators across low- to high-resourced settings: a scoping review. Disabil Rehabil Assist Technol. (2023) 18:67–88. 10.1080/17483107.2022.2037757 35436160 PMC7614122

[12] International Society of Wheelchair Professionals. Wheelchair Educators Package. Available online at: https://wep.iswp.org/ (Accessed December 9, 2023).

[13] Kirby RL Rushton PW Smith C. Wheelchair Skills Program. Available online at: https://wheelchairskillsprogram.ca/en/ (Accessed December 9, 2023).

[14] Burrola-Mendez Y Kirby RL Rushton PW Contepomi S Tawashy AE Kankipati P Psychometric properties of the international society of wheelchair Professionals’ basic manual wheelchair-service-provision knowledge test version 1 and development of version 2. PLoS One. (2023) 18:e0281584. 10.1371/journal.pone.0281584 36952447 PMC10035907

[15] Rushton PW Kirby RL Miller WC. Manual wheelchair skills: objective testing versus subjective questionnaire. Arch Phys Med Rehabil. (2012) 93:2313–8. 10.1016/j.apmr.2012.06.007 22728701 PMC3951990

[16] Giesbrecht E. Wheelchair skills test outcomes across multiple wheelchair skills training bootcamp cohorts. Int J Environ Res Public Health. (2021) 19(1):21. 10.3390/ijerph19010021 35010282 PMC8750881

[17] Rushton PW Daoust G. Wheelchair skills training for occupational therapy students: comparison of university-course versus “boot-camp” approaches. Disabil Rehabil Assist Technol. (2019) 14(6):595–601. 10.1080/17483107.2018.1486468 29996670

[18] ISWP. International Society of Wheelchairs Professionals. Available online at: https://wheelchairnetwork.org/trainer-recognition-certificates-awarded/?search_text=&iclocation=Ukraine&jclocation=&submit=FIND (Accessed December 9, 2023).

[19] Kirby RL Smith C Osmond D Moore SA Theriault CJ Sandila N. A remote-learning course can improve the subjective wheelchair-skills performance and confidence of wheelchair service providers: an observational cohort study. Disabil Rehabil Assist Technol. (2023) 19(4):1729–38. 10.1080/17483107.2023.2230259 37384537

[20] Worobey LA Kirby RL Cowan RE Dyson-Hudson TA Shea M Heinemann AW Using remote learning to teach clinicians manual wheelchair skills: a cohort study with pre- vs post-training comparisons. Disabil Rehabil Assist Technol. (2022) 17:752–9. 10.1080/17483107.2020.1804633 32809896 PMC8204376

[21] Giesbrecht E Carreiro N Mack CM. Improvement and retention of wheelchair skills training for students in entry-level occupational therapy education. Am J Occup Ther. (2021) 75:7501205160p1–p9. 10.5014/ajot.2021.040428 33399064

[22] Keeler L Kirby RL Parker K McLean KD Hayden JA. Effectiveness of the wheelchair skills training program: a systematic review and meta-analysis. Disabil Rehabil Assist Technol. (2019) 14:391–409. 10.1080/17483107.2018.1456566 29616832

[23] Kirby RL Worobey LA Cowan R Pedersen JP Heinemann AW Dyson-Hudson TA Wheelchair skills capacity and performance of manual wheelchair users with spinal cord injury. Arch Phys Med Rehabil. (2016) 97:1761–9. 10.1016/j.apmr.2016.05.015 27317867

[24] Mountain AD Kirby RL Smith C. The wheelchair skills test, version 2.4: validity of an algorithm-based questionnaire version. Arch Phys Med Rehabil. (2004) 85:416–23. 10.1016/S0003-9993(03)00427-1 15031827

[25] Rushton PW Smith EM Miller WC Kirby RL Daoust G. Reliability and responsiveness of the self- efficacy in assessing, training and spotting wheelchair skills (SEATS) outcome measure. Disabil Rehabil Assist Technol. (2019) 14:250–4. 10.1080/17483107.2018.1428370 29385845

[26] Layton N Bell D Borg J Steel E Maclachlan M Tebbutt E Assistive technology as a pillar of universal health coverage: qualitative analysis of stakeholder responses to the world health assembly resolution on assistive technology. Disabil Rehabil Assist Technol. (2020) 15:825–31. 10.1080/17483107.2020.1774929 32594831

[27] Kumurenzi A Richardson J Thabane L Kagwiza J Urimubenshi G Hamilton L Effectiveness of interventions by non-professional community-level workers or family caregivers to improve outcomes for physical impairments or disabilities in low resource settings: systematic review of task-sharing strategies. Hum Resour Health. (2023) 21:48. 10.1186/s12960-023-00831-7 37344907 PMC10286375

